# Cancer du poumon au Maroc Oriental: où en sommes-nous?

**DOI:** 10.11604/pamj.2019.34.177.19934

**Published:** 2019-12-05

**Authors:** Karam Yahya Belmokhtar, Mariam Tajir, Redouane Boulouiz, Amal Bennani, Sami Aziz Brahmi, Ihsan Alloubi, Hatim Kouismi, Imane Kamaoui, Imane Skiker, Said Afqir, Naima Abda, Mohammed Bellaoui, Loubna Mezouar

**Affiliations:** 1 Genetics Unit, Faculty of Medicine and Pharmacy of Oujda, University Mohammed Premier, Oujda, Morocco; 2 Faculty of Medicine and Pharmacy of Oujda, University Mohammed Premier, Mohammed VI University Hospital, Oujda, Morocco; 3 Hassan II Oncology Center, Oujda, Morocco

**Keywords:** Cancer du poumon, epidémiologie, tabagisme, cannabis, test moléculaire, Maroc Oriental, Lung cancer, epidemiology, smoking, cannabis, molecular test, Eastern Morocco

## Abstract

**Introduction:**

Le cancer du poumon est le cancer le plus fréquent chez l'homme au Maroc Oriental. Nous présentons ici le premier rapport sur les caractéristiques cliniques, pathologiques et thérapeutiques du cancer du poumon au Maroc Oriental.

**Méthodes:**

Etude rétrospective sur 738 patients diagnostiqués avec un cancer du poumon au niveau du Centre d'Oncologie Hassan II entre octobre 2005 et décembre 2014.

**Résultats:**

Parmi les cas étudiés, 671 étaient des hommes et 67 des femmes. Les fumeurs représentaient 95,01% des cas chez l'homme et 1,54% chez la femme. L'âge moyen au diagnostic était de 59,1 ± 11,9 ans. La majorité des patients (97%) étaient diagnostiqués à des stades avancés de la maladie. Parmi les 227 patients atteints d'adénocarcinome en stade avancé, le test moléculaire a été réalisé chez seulement 4 patients. En plus, aucun patient de notre série n'a bénéficié d'un traitement par la thérapie ciblée. Dans cette série, 20,46% des patients avaient moins de 50 ans. Comparé aux patients âgés de 50 ans et plus, les patients âgés moins de 50 ans présentaient une consommation de cannabis plus élevé (p<0,001) et un pourcentage d'adénocarcinome plus important (p<0,01), par contre, ils avaient une consommation tabagique moins importante (p<0,001), et un pourcentage plus faible de carcinome epidermoïde (p<0,01) et de CPC (p<0,05).

**Conclusion:**

Contrairement aux pays occidentaux, le cancer du poumon est diagnostiqué tardivement, touche une population plus jeune au Maroc Oriental et l'accès aux tests moléculaires est très faible. Ces résultats justifient la nécessité d'établir un programme efficace de lutte contre le cancer du poumon au Maroc Oriental et faciliter l'accès aux tests moléculaires et aux nouveaux outils thérapeutiques.

## Abstract (en)

**Introduction:**

Lung cancer is the most common cancer in men living Eastern Morocco. We here present the first report on the clinical, pathological and therapeutic features of lung cancer in Eastern Morocco.

**Methods:**

We conducted a retrospective study of 738 patients diagnosed with lung cancer at the Hassan II, Oncology Center between October 2005 and December 2014.

**Results:**

Among the cases studied, 671 patients were men and 67 women; 95.01% of men and 1.54% of women were smokers. The average age of patients was 59.1 ± 11.9 years. Most patients (97%) were diagnosed at advanced stage disease. Only 4 out of 227 patients with advanced adenocarcinoma underwent molecular test. In addition, no patient in our series received targeted therapy. In this series, 20.46% of patients had less than 50 years. Compared to patients aged 50 years and older, cannabis consumption was higher (p<0.001) in patients less than 50 years and as well as a higher rate of adenocarcinoma (p<0.01). By contrast, in these patients, tobacco consumption was lower (p<0.001) as well as the rate of squamous cell carcinoma (p<0.01) and small cell cancer (p<0.05).

**Conclusion:**

Unlike Western countries, in Eastern Morocco lung cancer is diagnosed late, affects younger people and access to molecular tests is still very limited. These results justify the need to implement effective programs against lung cancer as well as to facilitate access to molecular tests and new therapeutic tools in Eastern Morocco.

## Introduction

Le cancer du poumon est le cancer le plus fréquent dans le monde et représente la première cause de décès par cancer [1,2]. L'incidence de la maladie ne cesse d'augmenter et le nombre de décès rapporté dépasse largement celui du cancer du sein, prostate et colorectal réunis [3,4]. Le pronostic de la maladie reste péjoratif; le taux de survie à 5 ans varie de 6 à 14% chez les hommes et de 7 à 18% chez les femmes [5]. Le principal facteur de risque est le tabagisme, responsable de 90% des cas avec une relation exposition-effet [6]. Les cancers du poumon sont classés histologiquement en deux catégories: les carcinomes non à petites cellules (CNPC) incluant principalement les adénocarcinomes, les carcinomes épidermoïdes, les carcinomes neuroendocrines à grande cellules et les carcinoïdes d'une part, et les carcinomes à petites cellules (CPC) d'autre part. Grace aux nouvelles connaissances apportées par des études multidisciplinaires, la classification anatomo-pathologique a été modifiée récemment [7]. En absence d'un registre national du cancer du poumon au Maroc, très peu de données sont disponibles sur l'état des lieux de cette pathologie. Ainsi, il est difficile de développer des programmes de lutte contre ce cancer. Dans cette étude, nous présentons pour la première fois les caractéristiques épidémiologiques, clinico-pathologiques et thérapeutiques du cancer bronchique primitif au niveau du Maroc Oriental.

## Méthodes

**Conception de l'étude et collecte de données:** il s'agit d'une étude rétrospective et descriptive réalisée au Centre Régional d'Oncologie-Hassan II (CRO) entre octobre 2005 et décembre 2014. Le CRO, créé en octobre 2005, est un centre spécialisé dans le traitement du cancer et il est le seul centre d'oncologie dans la région du Maroc Oriental. Le Maroc Oriental est localisé au nord-est du royaume du Maroc. Pendant la période d'étude, la région du Maroc Oriental a été composée d'une préfecture (Oujda) et six provinces (Nador, Berkane, Driouch, Figuig, Taourirt et Jerada). Tous les patients résidant dans la région du Maroc Oriental et présentant un cancer du poumon confirmé histologiquement ou cytologiquement ont été inclus dans cette étude. Les patients ayant un cancer du poumon secondaire, ou n'ayant pas de preuve histologique ont été exclus de l'étude. Les patients ne résidant pas dans le Maroc Oriental ou pour qui le lieu de résidence n'a pas été spécifié ont également été exclus de l'étude. Les données ont été saisies sur Excel.

**Analyse des données:** l'analyse statistique des données a été faite sur le programme SPSS version 21.0. Le test Chi^2^ de Pearson et le test exact de Fisher ont été utilisés pour étudier la relation entre les variables catégorielles. Alors que le Test-T et l'analyse de variance (ANOVA) ont été utilisés pour analyser la relation entre les variables catégorielles et continues. Le seuil de signification a été fixé à p<0,05 pour tous les tests statistiques.

**Considérations éthiques:** un protocole de l'étude a été soumis et approuvé par le Comité d’éthique pour la recherche biomédicale de la Faculté de Médecine et de Pharmacie de Casablanca sous le numéro 41/14. L'autorisation de traitement des données à caractère personnel a été obtenue auprès de la commission nationale de contrôle de la protection des données personnelles sous le numéro A-RS-280/2014. Tout au long de l'étude, les dossiers et renseignements des patients ont été conservés de manière anonyme et le respect de la confidentialité des données a été assuré.

## Résultats

**Caractéristiques générales du cancer du poumon au Maroc Oriental:** sur la période de 2005 à 2014, 738 patients ayant un cancer du poumon ont été enregistrés au CRO, dont 671 hommes (91%) et 67 femmes (9%). L'âge moyen des patients au moment du diagnostic était de 59,13 ± 11,93 ans (âge médian de 58 ans). Chez les hommes, il était de 59,08 ± 11,96 ans (âges extrêmes allant de 15 et 91 ans) et chez les femmes il était de 59,58 ± 11,72 ans (âges extrêmes allant de 33 et 84 ans). L'analyse statistique a montré qu'il n'y a pas de différence significative de l'âge moyen entre les deux sexes (Tableau 1). La répartition des patients selon l'âge a montré que 20,46% des cancers du poumon ont été observés chez les patients âgés de moins de 50 ans, 33,6% chez la tranche d'âges 50-59 ans, 22,9% chez la tranche d'âges 60-69 ans et 23,04% chez les patients âgés de 70 ans et plus (Tableau 1). Chez les hommes, les fumeurs représentaient 95,01% des cas, alors que chez les femmes, les fumeuses représentaient seulement 1,54% des cas et cette différence est significative (p<0,001). La consommation tabagique moyenne dans notre population était de 37,58 ± 20,85 paquet-années avec une durée de consommation moyenne de 30,92 ± 11,18 années (Tableau 1). Les antécédents les plus rencontrés étaient le diabète (4,61%), les néoplasies autres que le cancer du poumon (3,93%) et l'hypertension artérielle (HTA, 2,44%). L'HTA et le diabète étaient beaucoup plus fréquents chez les femmes que chez les hommes et cette différence entre les deux sexes est significative (p≤0.001 pour HTA, p<0,01 pour le diabète) (Tableau 1).

**Tableau 1:** Caractéristiques générales du cancer du poumon au Maroc Oriental (n=738)

	Total	Hommes	Femmes	*p*
	n=738	n=671	n=67	
**Age moyen (ans ± SD)**	59,13 ± 11,93	59,08 ± 11,96	59,58 ± 11,72	NS
**Age médian (ans)**	58	58	59	
**Age par tranches**				NS
< 50 ans (N (%))	151 (20,46)	137 (20,42)	14 (20,90)	
50 - 59 ans (N (%))	248 (33,60)	227 (33,83)	21 (31,34)	
60 - 69 ans (N (%))	169 (22,90)	154 (22,95)	15 (22,39)	
> 70 ans (N (%))	170 (23,04)	153 (22,80)	17 (25,37)	
Tabagisme (n*=686)				<0,001
Fumeurs (N (%))	591 (86,15)	590 (95,01)	1 (1,54)	
Non-fumeurs (N (%))	95 (13,85)	31 (4,99)	64 (98,46)	
Consommation tabagique (PA ± SD)	37,58 ± 20,85	-	-	
Durée de consommation (ans ± SD)	30,92 ± 11,18	-	-	
**Antécédents**				
HTA (N (%))	18 (2,44)	11 (1,64)	7 (10,45)	≤0,001
Diabète (N (%))	34 (4,61)	26 (3,87)	8 (11,94)	<0,01
BPCO (N (%))	5 (0,68)	5 (0,74)	0 (0)	NS
Tuberculose (N (%))	9 (1,22)	9 (1,34)	0 (0)	NS
Néoplasies (N (%))	29 (3,93)	25 (3,73)	4 (5,97)	NS

NS: non significatif; n*: Le nombre de cas chez qui l’information concernant le facteur analysé a été retrouvé parmi les 738 cas; PA: paquet-années ; HTA : hypertension artérielle; BPCO: Bronchopneumopathie chronique obstructive

**Caractéristiques clinico-pathologiques du cancer du poumon au Maroc Oriental:** dans notre série, les symptômes les plus rencontrés étaient la toux (49,59% des cas), les douleurs thoraciques (45,39%), l'hémoptysie (32,11%), la dyspnée (30,89%), et l'altération de l'état général (27,37%). La dyspnée était plus fréquente chez les femmes et cette différence entre les deux sexes est significative (Tableau 2). La durée moyenne de la symptomatologie (délai entre l'apparition des symptômes et la prise en charge) de la population étudiée était de 4,84 ± 6,98 mois. Nous n'avons pas noté de différence significative entre les hommes et les femmes concernant la période de symptomatologie (Tableau 2). Dans notre étude, le signe clinique le plus fréquent était les adénopathies chez 62,83% des cas suivi respectivement des pleurésies (31,63%), des emphysèmes (18,37%), des extensions tumorales médiastinales (14,87%), des extensions tumorales pariétales (14,14%), de l'épaississement pleural (11,52%), des atélectasies (10,06%) et enfin des pneumothorax (1,46%) (Tableau 2). Il existe une différence significative entre les deux sexes dans la fréquence de l'atélectasie, la pleurésie et l'emphysème (Tableau 2). Dans notre série, la majorité des tumeurs ont été localisées dans le poumon droit (54,81%), suivi du poumon gauche (33,24%). Concernant les lobes pulmonaires atteints, le lobe supérieur droit était le plus touché (36,78%) suivi du lobe supérieur gauche (19,21%) (Tableau 2). Plusieurs métastases ont été observées: les métastases cérébrales (17,89%), pulmonaires (16,94%), hépatiques (16,67%), osseuses (15,04%), surrénales (14,77%) et pleurales (6,23%). En plus, d'autres métastases plus rares ont été enregistrées (1,63%, n=12) et concernaient les reins, le pancréas, la rate, la peau et le médiastin. Les métastases pleurales étaient plus fréquentes chez les femmes que chez les hommes (17,19% contre 5,07%, p<0.001) (Tableau 2).

**Tableau 2:** Caractéristiques clinico-pathologiques du cancer du poumon au Maroc Oriental (n=738)

	Total	Hommes	Femmes	*p*
**Symptômes**				
Toux (N (%))	366 (49,59)	332 (49,48)	34 (50,75)	NS
Douleurs thoraciques (N (%))	335 (45,39)	311 (46,34)	24 (35,82)	NS
Hémoptysie (N (%))	237 (32,11)	221 (32,94)	16 (23,88)	NS
Dyspnée (N (%))	228 (30,89)	199 (29,66)	29 (43,28)	< 0,05
AEG (N (%))	202 (27,37)	183 (27,27)	19 (28,36)	NS
**Durée de symptomatologie (mois)**	4,84 ± 6,98	4,75 ± 6,91	5,96 ± 7,65	NS
Signes cliniques (n*=686)				
Adénopathies (N (%))	431 (62,83)	392 (63,02)	39 (60,94)	NS
Métastases (N (%))	263 (38,39)	236 (37,94)	27 (42,19)	NS
Pleurésie (N (%))	217 (31,63)	184 (29,58)	33 (51,56)	< 0,001
Emphysème (N (%))	126 (18,37)	125 (20,10)	1 (1,56)	< 0,001
Extension médiastinale (N (%))	102 (14,87)	94 (15,11)	8 (12,50)	NS
Extension pariétale (N (%))	97 (14,14)	93 (14,95)	4 (6,25)	NS
Epaississement pleural (N (%))	79 (11,52)	69 (11,09)	10 (15,63)	NS
Atélectasie (N (%))	69 (10,06)	55 (8,84)	14 (21,88)	≤0,001
Pneumothorax (N (%))	10 (1,46)	8 (1,29)	2 (3,13)	NS
Localisation des tumeurs (n*=686)				
Poumon droite (N (%))	376 (54,81)	347 (55,79)	29 (45,31)	NS
Poumon gauche (N (%))	228 (33,24)	202 (32,48)	26 (40,63)	
Atteinte bilatérale (N (%))	5 (0,73)	4 (0,64)	1 (1,56)	
Médiastin (N (%))	22 (3,21)	22 (3,54)	0 (0)	
Sites pulmonaires atteints (n*=609)				
Lobe supérieur droit (N (%))	224 (36,78)	209 (37,79)	15 (26,79)	NS
Lobe supérieur gauche (N (%))	117 (19,21)	106 (19,17)	11 (19,64)	NS
Lobe inferieur droit (N (%))	63 (10,35)	60 (10,85)	3 (5,36)	NS
Lobe inferieur gauche (N (%))	62 (10,18)	52 (9,40)	10 (17,86)	< 0.05
Lobe moyen droit (N (%))	31 (5,09)	24 (4,34)	7 (12,50)	<0,05
**Métastases**				
Cérébrale (N (%))	132 (17,89)	120 (17,88)	12 (17,91)	NS
Pulmonaire (N (%))	125 (16,94)	112 (16,69)	13 (19,40)	NS
Hépatique (N (%))	123 (16,67)	111 (16,54)	12 (17,91)	NS
Osseuse (N (%))	111 (15,04)	105 (15,65)	6 (8,96)	NS
Surrénale (N (%))	109 (14,77)	104 (15,50)	5 (7,46)	NS
Pleurale (N (%))	46 (6,23)	34 (5,07)	12 (17,91)	< 0,001
Autres (N (%))	12 (1,63)	10 (1,49)	2 (2,99)	NS

n*: Le nombre de cas chez qui l’information concernant le facteur analysé a été retrouvé parmi les 738 cas; NS: non significatif

**Caractéristiques anatomopathologiques et thérapeutiques du cancer du poumon au Maroc Oriental:** le Tableau 3 résume la répartition des cas de cancer du poumon suivant les différents types histologiques. Dans notre série, les carcinomes à petites cellules (CPC) représentaient 12,47% des cas et les carcinomes non à petites cellules (CNPC) 76,69%. L'adénocarcinome (46,48%) et le carcinome épidermoïde (26,69%) étaient les types histologiques les plus fréquents. Les carcinomes à grandes cellules représentaient 1,90% des cas, les carcinomes neuroendocriniens à grandes cellules (CNEGC) 0,54%, les carcinomes muco-epidermoïdes 0,27%, les carcinomes adenosquameux 0,41%, les carcinoïdes 0,13% et les carcinomes sarcomatoïdes 0,27%. Les autres types histologiques ne représentaient que 3,66%. Nous avons trouvé une différence significative de la répartition des adénocarcinomes et carcinomes epidermoïdes entre les deux sexes. En effet, l'adénocarcinome était plus fréquent chez la femme (64,17% contre 44,71%, p<0,005), au contraire, le carcinome epidermoïde était environ 3 fois plus fréquent chez l'homme (28,32% contre 10,45% respectivement, p<0,005) (Tableau 3). Les types histologiques les plus fréquents ont montré une différence significative (p≤0,001) selon l'âge au diagnostic. L'adénocarcinome a été associé à un âge plus jeune (57,70 ± 11,48 ans) en comparaison avec le carcinome épidermoïde (61,93 ± 12,02 ans) et le CPC (60,70 ± 11,38 ans) (Tableau 3). La majorité de nos patients avaient un stade avancé du cancer du poumon à la présentation (97% des stades III et IV). En effet, dans le cas des CNPC, 90,91% étaient en stade IV, 6,31% en stade III. A l'instar des CNPC, la majorité des CPC étaient d'emblée à l'état diffus (96,49%). Il n'y avait aucune différence significative entre l'homme et la femme concernant la répartition des stades de la maladie (Tableau 3). Concernant les modalités thérapeutiques, la chimiothérapie était le traitement le plus fréquent (64,77%), suivi de la radiothérapie (30,89%), des soins palliatifs (14,12%) et de la chirurgie (5,28%). Pour les modalités thérapeutiques, il n'y avait pas de différence significative dans les proportions entre l'homme et la femme (Tableau 3).

**Tableau 3:** Caractéristiques anatomopathologiques et thérapeutique du cancer du poumon au Maroc Oriental (n=738)

	Total	Hommes	Femmes	*p*
**Types histologiques**				
Carcinome à petites cellules_(N (%))	92 (12,47)	87 (12,97)	5 (7,46)	NS
*Age au diagnostic (ans ± SD)*	60,70 ± 11,38	-	-	
Carcinome non à petites cellules_(N (%))	566 (76,69)	514 (76,60)	52 (77,61)	NS
Adénocarcinome (N (%))	343 (46,48)	300 (44,71)	43 (64,17)	<0,005
*Age au diagnostic (ans ± SD)*	57,70 ± 11,48	-	-	
Carcinome épidermoïde (%)	197 (26,69)	190 (28,32)	7 (10,45)	<0,005
*Age au diagnostic (ans ± SD)*	61,93 ± 12,02	-	-	
Carcinome à grandes cellules (N (%))	14 (1,90)	14 (2,09)	0 (0)	NS
CNEGC (N (%))	4 (0,54)	4 (0,60)	0 (0)	NS
Carcinome muco-epidermoïde (N (%))	2 (0,27)	1 (0.15)	1 (1,49)	NS
Carcinome adenosquameux (N (%))	3 (0,41)	3 (0,45)	0 (0)	NS
Carcinoïde (N (%))	1 (0,13)	1 (0,15)	0 (0)	NS
Carcinome sarcomatoïde (N (%))	2 (0,27)	1 (0,15)	1 (1,49)	NS
Carcinomes SAI (N (%))	53 (7,18)	47 (7,00)	6 (8,95)	NS
Autres* (N (%))	27 (3,66)	23 (3,43)	4 (5,97)	NS
**Stades**				
All (n*=501)				NS
I + II (N (%))	15 (3,00)	14 (3,03)	1 (2,56)	
III + IV (N (%))	486 (97,00)	448 (96,97)	38 (97,44)	
CNPC (n*=396)				NS
I (N (%))	4 (1,01)	3 (0,82)	1 (3,12)	
II (N (%))	7 (1,77)	7 (1,92)	0 (0)	
III (N (%))	25 (6,31)	25 (6,87)	0 (0)	
IV (N (%))	360 (90,91)	329 (90,39)	31 (96,88)	
CPC (n*=57)				NS
Localisé (N (%))	2 (3,51)	2 (3,64)	0 (0)	
Diffus (N (%))	55 (96,49)	53 (96,36)	2 (100)	
**Modalités thérapeutiques**				
Chirurgie (N (%))	39 (5,28)	33 (4,94)	6 (8,95)	NS
Chimiothérapie (N (%))	478 (64,77)	431 (64,23)	47 (70,15)	NS
Radiothérapie (N (%))	228 (30,89)	211 (31,44)	17 (25,37)	NS
Chimio-radiothérapie (N (%))	178 (24,12)	163 (24,29)	15 (22,39)	NS
Soins palliatifs (N (%))	104 (14,09)	93 (13,86)	11 (16,42)	NS

NS: non significatif, CNPC: Carcinome non à petites cellules, CPC: Carcinome à petites cellules, CNEGC: Carcinome neuroendocrine à grandes cellules, SAI: Sans autres indication; Autres: liposarcome pulmonaire, sarcome pulmonaire, lymphome pulmonaire, plasmocytome pulmonaire et les proliférations malignes sans autre indication; n*: Le nombre de cas chez qui l’information concernant le facteur analysé a été retrouvé parmi les 738 cas

**Tests moléculaires du cancer du poumon au Maroc Oriental:** dans notre série, parmi les 227 patients atteints d'adénocarcinome en stade avancé, le test moléculaire a été réalisé chez seulement 4 patients par analyse de l'EGFR et/ou KRAS. En effet, chez une patiente, l'analyse des exons 18-21 du gène EGFR et des exons 12-13 du gène KRAS a mis en évidence une délétion de l'exon 19 de l'EGFR et l'absence de mutations au niveau des exons étudiés du gène KRAS. Chez les trois autres patients seule l'analyse des exons 18-21 du gène EGFR a été demandée et s'est avérée négative dans les trois cas.

**Le cancer du poumon chez les patients âgés moins de 50 ans et ceux âgés de 50 ans et plus au Maroc Oriental:** la comparaison des habitudes tabagiques et les types histologiques entre les patients âgés moins de 50 ans et ceux âgés de 50 ans et plus est représenté dans le Tableau 4. Ces deux groupes d'âges ont présenté une différence significative selon la consommation tabagique, la durée de consommation et la consommation de cannabis. En effet, les patients âgés moins de 50 ans ont été caractérisées par une consommation tabagique et une durée de consommation plus faibles (p<0,001) d'une part, et une consommation de cannabis plus importante (p<0,001) d'autre part par rapport aux patients âgés de 50 ans et plus. De la même façon, une différence significative a été trouvée entre les deux groupes d'âges concernant le pourcentage des types histologiques les plus fréquents. En effet, par rapport aux patients âgés de 50 ans et plus, les patients âgés moins de 50 ans présentaient un pourcentage d'adénocarcinome plus élevé (p<0,01) en revanche ils avaient un pourcentage plus faible de carcinome epidermoïde (p<0,01) et de CPC (p<0,05).

**Tableau 4:** Comparaison des habitudes tabagiques et types histologiques entre les patients âgés moins de 50 ans et ceux âgés de 50 ans et plus au Maroc Oriental

	Moins de 50 ans (n=151)	50 ans et plus (n=587)	*p*
Tabagisme (n*=141 et 545 respectivement)			
Fumeurs (N (%))	122 (86,53)	469 (86,05)	NS
Non-fumeurs (N (%))	19 (13,47)	76 (13,95)	
Consommation tabagique (PA ± SD)	26,60 ± 13,56	40,64 ± 21,49	<0,001
Durée de consommation (ans ± SD)	23,48 ± 7,87	32,99 ± 11,10	<0,001
**Consommation du cannabis (N (%))**	27 (17,88)	33 (5,62)	<0,001
**Types histologiques**			
Adénocarcinome (N (%))	85 (56,29)	258 (43,95)	<0,01
Carcinome épidermoïde (N (%))	27 (17,88)	170 (28,96)	<0,01
CPC (N (%))	11 (7,28)	81 (13,8)	<0,05

PA: paquet-années; CPC: carcinome à petites cellules; n*: Le nombre de cas chez qui l’information concernant le facteur analysé a été retrouvé parmi l’ensemble des cas ; NS: non-significatif

## Discussion

Le cancer du poumon est de loin le premier cancer en termes d'incidence et de mortalité avec 1,82 millions de nouveaux cas et 1,59 millions de décès par cancer du poumon en 2012 dans le monde [1]. Les estimations pour l'année 2018 sont alarmantes, l'incidence de la maladie aura atteint 2,1 millions de nouveaux cas avec une mortalité de 1,8 millions de décès [4]. L'incidence chez les hommes diminue tandis que celle des femmes augmente notamment dans les pays où le tabagisme féminin commence à prendre de l'ampleur. Au Maroc Oriental, le cancer du poumon est le premier cancer chez l'homme en termes d'incidence représentant 19% de l'ensemble des cancers [8]. En terme de prévalence, Elidrissi Errahhali *et al*. ont trouvé que chez les hommes, le cancer du poumon est le type de cancer le plus fréquent dans la majorité des régions du Maroc Oriental [9]. Cette situation du cancer du poumon au Maroc Oriental nous a poussés à réaliser cette étude pour mieux comprendre cette pathologie et déterminer les champs d'action pour réduire son fardeau sur la société. Sur les 738 cas de notre étude il y avait dix fois plus d'hommes (90,92%) que de femmes (9,08%). Cette prédominance masculine est similaire à celle observée dans les registres de Casablanca et de Rabat [10-12] ainsi que dans d'autres pays nord-africains [13,14]. Un sex-ratio élevé est classiquement observé dans les séries où le tabagisme féminin est moins important [15-20]. Dans notre série, la majorité des patients atteints de cancer du poumon étaient des fumeurs, ce qui suggère que le tabagisme est le facteur de risque principal du cancer du poumon dans la population du Maroc Oriental, surtout chez les hommes où le tabagisme a représenté 95,01% des cas. Il est important de noter que chez les femmes la majorité était non fumeuse (66 sur 67 cas), cela suggère qu'il y a probablement d'autres facteurs de risque responsables du cancer du poumon chez les femmes au Maroc Oriental. En effet, plusieurs études ont impliqué d'autres facteurs de risque dans le cancer du poumon comme le tabagisme passif, les expositions professionnelles et environnementales à des agents cancérigènes, la tuberculose, l'infection par le papillomavirus humain, des facteurs hormonaux, des facteurs génétiques etc. [21]. Les comorbidités associées au cancer du poumon peuvent compliquer la prise en charge thérapeutique ou encore limiter certaines chimiothérapies [22]. Dans notre étude, les comorbidités associées au cancer du poumon étaient présentes chez une minorité de nos patients, ce qui est différent par rapport à la situation dans d'autres études [16,20]. D'autre part, dans notre étude, nous avons noté que les comorbidités associées au cancer du poumon sont plus fréquentes chez les femmes que chez les hommes, ce qui est en accord avec les données rapportées dans d'autres études [20].

Dans notre série les symptômes étaient dominés par la toux, les douleurs thoraciques, l'hémoptysie et la dyspnée, Cependant la fréquence de ces symptômes est supérieure à celle rapportée dans la littérature [13,17,18,20,23,24]. Dans notre série, la majorité des tumeurs sont localisées dans le poumon droit et plus particulièrement le lobe supérieur droit, ce qui est en accord avec plusieurs travaux publiés antérieurement [13,17,23,25,26]. Dans notre étude l'adénocarcinome est le type histologique le plus fréquent chez les hommes et les femmes, ce qui en accord avec les données du registre de Rabat et d'autres études [12,15,20,27,28]. Cependant, d'autres études le place en 2^ème^ position en faveur d'une prédominance du carcinome epidermoïde [10,11,13,16,19,23,25,26,29,30]. Concernant le CPC, il occupe la troisième position après l'adénocarcinome et le carcinome epidermoïde, et il est plus fréquent chez les hommes que chez les femmes. Cette différence de répartition des types histologiques entre les deux sexes peut être expliquée par la susceptibilité vis-à-vis du tabac [31]. En effet, dans les études rapportant un tabagisme féminin important, la fréquence des CPC était plus élevée chez les femmes que chez les hommes [15,19]. Dans notre série, la majorité des patients atteints de CNPC ou de CPC étaient diagnostiqués à des stades avancés de la maladie (97%), ce qui est nettement supérieur à ce qui a été rapporté dans d'autres études [13,16,20]. Ces chiffres sont alarmants et incite à mettre en place un programme efficace de lutte contre le cancer du poumon au Maroc Oriental. Notre étude a révélé que le cancer du poumon au Maroc Oriental touche des patients âgés de 15 à 91 ans avec un âge moyen au diagnostic de 59,1 ans. Nos résultats sont similaires aux données sur les populations marocaines de Casablanca et de Rabat, et dans l'Ouest algérien [10-13,32,33]. En revanche, dans d'autres pays le cancer du poumon survient à un âge plus avancé [3,5,10,12,14-17,19,26,30,34]. Ainsi, contrairement aux pays occidentaux, le cancer du poumon au Maroc Oriental touche une population plus jeune. Ce phénomène de jeune âge observé est probablement dû au fait que la population est plus jeune que la population européenne.

Mais d'autres facteurs peuvent être impliqués, notamment génétiques et environnementaux. Par exemple, la consommation de cannabis plus importante observée chez les patients âgés moins de 50 ans pourrait être liée au cancer du poumon chez cette population. En effet, dans une étude meta-analyse le cannabis a été associé avec un risque plus élevé de développer le cancer du poumon [34,35]. Toutefois, d'autres études sont nécessaires pour comprendre la survenue du cancer du poumon à un âge plus jeune au Maroc Oriental. Il est important de noter que chez les femmes, la majorité était non fumeuse (66 sur 67 cas), ce qui suggère qu'il y a probablement d'autres facteurs de risque responsables du cancer du poumon chez les femmes au Maroc Oriental. En effet, plusieurs études ont impliqué d'autres facteurs de risque dans le cancer du poumon chez les non-fumeurs comme le tabagisme passif, les expositions professionnelles et environnementales à des agents cancérigènes, la tuberculose, l'infection par le papillomavirus humain, des facteurs hormonaux, des facteurs génétiques etc. [21]. Une étude étiologique chez les non-fumeuses nous semble très utile pour identifier les facteurs de risque impliqués. L'émergence des inhibiteurs des tyrosines kinases tel que Gefitinib, Erlotinib, Afatinib et Osimertinib a permis une amélioration de la survie et de la qualité de vie des patients métastatiques. Par conséquent, la recherche des mutations du gène EGFR est systématiquement recommandée chez les patients ayant un adénocarcinome en stade IV [36]. Cependant, nos résultats montrent que l'analyse par biologie moléculaire des mutations du gène EGFR a été réalisée chez seulement 1,76% des patients ayant un adénocarcinome en stade IV. Ce faible pourcentage de tests moléculaires réalisés peut être expliqué par le coût élevé de ces tests qui ne sont pas pris en charge par l'assurance maladie et par le fait que la majorité des patients au Maroc Oriental n'ont pas les moyens de les payer [8].

## Conclusion

Cette étude rapporte, pour la première fois, les caractéristiques épidémiologiques, clinico-pathologiques et thérapeutiques du cancer du poumon au Maroc Oriental. Le tabac reste le principal facteur de risque impliqué dans la survenue du cancer du poumon. La majorité de nos patients avaient un stade avancé du cancer du poumon lors du diagnostic, de plus, peu de patients ont eu accès aux tests moléculaires et aucun d'eux n'a bénéficié d'une thérapie ciblée. Nos résultats justifient ainsi la nécessité d’établir un programme efficace de lutte contre le cancer du poumon au Maroc Oriental et faciliter l'accès aux tests moléculaires et aux nouveaux outils thérapeutiques.

### Etat des connaissances actuelles sur le sujet

- Le cancer du poumon est le cancer le plus fréquent dans le monde et représente la première cause de décès par cancer;
- Le principal facteur de risque est le tabagisme.

### Contribution de notre étude à la connaissance

- La première description du profil du cancer du poumon au Maroc Oriental sur une large série;
- Le cancer du poumon est diagnostiqué tardivement et touche une population plus jeune au Maroc Oriental;
- Les tests moléculaires sont quasiment absents dans la prise en charge des patients métastatiques.

## Conflits d’intérêts

Les auteurs ne déclarent aucun conflit d'intérêts.

## Remerciements

Nous remercions sincèrement le Pr. Tijani El Harroudi qui nous a permis l'accès aux dossiers cliniques. Nos remerciements s'adressent également à tout le personnel du Centre d'Oncologie Hassan II d'Oujda qui nous a assuré les conditions favorable pour l'étude des dossiers. Nous remercions également le Pr. A. Azzouzi et tout le personnel administratif de la Faculté de Médecine et de Pharmacie d'Oujda pour leur soutien et leurs encouragements précieux tout au long de ce travail. Nous tenons à remercier également Mr. Ihab Belmokhtar pour son aide dans la collecte et l'analyse des données.
